# Peptide Self-Assembly Is Linked to Antibacterial, but Not Antifungal, Activity of Histatin 5 Derivatives

**DOI:** 10.1128/mSphere.00021-20

**Published:** 2020-04-01

**Authors:** Lee Schnaider, Alexander Rosenberg, Topaz Kreiser, Sofiya Kolusheva, Ehud Gazit, Judith Berman

**Affiliations:** a Department of Molecular Microbiology and Biotechnology, George S. Wise Faculty of Life Sciences, Tel Aviv University, Tel Aviv, Israel; b Ilse Katz Institute for Nanotechnology, Ben Gurion University of the Negev, Beer Sheva, Israel; c Department of Materials Science and Engineering, Iby and Aladar Fleischman Faculty of Engineering, Tel Aviv University, Tel Aviv, Israel; Hackensack Meridian Health Center for Discovery and Innovation

**Keywords:** antimicrobial peptides, histatins, membrane interactions, self-assembly

## Abstract

The rise of multidrug-resistant pathogens has awakened interest in new drug candidates such as antimicrobial peptides and their derivatives. Recent work suggests that some antimicrobial peptides have the ability to self-assemble into ordered amyloid-like nanostructures which facilitate their antibacterial activity. Here, we evaluate a histatin-based antimicrobial peptide, and its self-assembling derivative, in the interplay between self-assembly, membrane interactions, and antibacterial and antifungal activities. We demonstrate substantial membrane targeting by both peptides, as well as mechanistic insights into this mode of action, which correlates to their antifungal activity and is not affected by their self-assembling state. The ability to self-assemble does, however, significantly affect peptide antibacterial activity against both Gram-negative and Gram-positive bacteria. These results are surprising and hint at important distinctions between antifungal and antibacterial peptide activities in prokaryotes and eukaryotic microbes.

**IMPORTANCE** Antimicrobial peptides are important modulators of host defense against bacterial, fungal, and viral pathogens in humans and other multicellular organisms. Two converging paradigms point to a link between antimicrobial peptides that self-assemble into amyloid-like nanoassemblies and classical amyloidogenic peptides that often have potent broad-spectrum antimicrobial activity, suggesting that antimicrobial and amyloidogenic peptides may represent two sides of the same coin. Here, we asked if the ability of an antifungal peptide to self-assemble affects its antifungal or antibacterial activity. We found that modifications of classical antifungal peptide derivative allowed it to self-assemble and did not alter its antifungal activity, and yet self-assembly substantially increased the antibacterial activity of the peptide. These results support the idea that peptide self-assembly can enhance antibacterial activities and emphasize a distinction between the action of antifungal peptides and that of antibacterial peptides. Accordingly, we suggest that the possible generality of this distinction should be widely tested.

## Abstract (precis)

Antimicrobial peptides are important modulators of host defense against bacterial, fungal, and viral pathogens in humans and other multicellular organisms. Two converging paradigms point to a link between antimicrobial peptides that self-assemble into amyloid-like nanoassemblies and classical amyloidogenic peptides that often have potent broad-spectrum antimicrobial activity, suggesting that antimicrobial and amyloidogenic peptides may represent two sides of the same coin. Here, we asked if the ability of an antifungal peptide to self-assemble affects its antifungal or antibacterial activity. We found that modifications of classical antifungal peptide derivative allowed it to self-assemble and did not alter its antifungal activity, and yet self-assembly substantially increased the antibacterial activity of the peptide. These results support the idea that peptide self-assembly can enhance antibacterial activities and emphasize a distinction between the action of antifungal peptides and that of antibacterial peptides. Accordingly, we suggest that the possible generality of this distinction should be widely tested.

## INTRODUCTION

Rapid growth in the number and prevalence of multidrug-resistant bacteria, coupled with a 30-year void in the introduction of new antibiotics into clinical practice, underlies the need for new antibacterial agents, especially those based on unique mechanisms of action (1–4). Antimicrobial peptides (AMPs), a growing class of membrane-interacting antimicrobial agents, are active against a broad spectrum of microorganisms (5–8), and their synthetic analogs and mimetics constitute a potential source of new antimicrobial therapeutics (9, 10).

Candida albicans is the most prevalent cause of oral candidiasis and of candidal infections in general (11, 12). Resistance and tolerance to antifungal drugs are serious concerns for C. albicans as well as for several emerging non-*albicans Candida* species (13–15). The first-line defense against oral candidiasis involves the use of histatins, a collection of at least 12 salivary histidine-rich antimicrobial peptides (16–18). Histatin 5 is among the most extensively studied and most effective of these against *Candida* infections (18–22). Several histatin 5 derivatives with increased potency and stability have been developed by amphipathic optimization (23–25). Histatin 5 derivatives dhvar1 and dhvar4 are membrane active and able to dissipate cytoplasmic transmembrane potential, as well as uncouple the respiration of isolated mitochondria (23–26). While histatin 5 may have other cytoplasmic targets (24, 27), these membrane-active histatin derivatives elicit a response similar to that of classical pore-forming antimicrobial peptides (24).

Interestingly, several classical antimicrobial peptides self-assemble in a manner that retains and/or augments their activity (28–34). Furthermore, some amyloidogenic peptides have potent antimicrobial activities (35–39), suggesting that these two classes of peptides may not be as distinct as previously thought (reviewed previously [40–45]). We recently found that self-assembly of minimal amyloid models augments their antibacterial activity (46). Furthermore, impressive intrinsic antibacterial activity (47–50) has been detected in several studies using aromatic-amino-acid-based self-assembling building blocks (47–50). Whether self-assembly has a role in antifungal peptide activity is not well established.

Here, we investigated the relationship between peptide self-assembly, membrane interactions, and antibacterial and antifungal activities using dhvar2 (KRLFKELLFSLRKY), a well-characterized histatin 5 derivative, as a model. We identified a single-amino-acid mutation, L7F (KRLFKEFLFSLRKY), which is required to facilitate peptide self-assembly into ordered nanostructures. This mutation does not change the amphipathic nature of the peptide but drastically affects its self-assembly potential. The membrane-interacting capabilities of dhvar2 as well as L7F correlated positively with their antifungal activity. Surprisingly, peptide self-assembly did not affect overall antifungal activity but significantly affected antibacterial activity against both Escherichia coli and Staphylococcus epidermidis. These results highlight a new distinction between self-assembly and AMP activity in bacteria versus fungi, although the degree to which this is a general principle remains to be tested.

## RESULTS

### Self-assembly of a histatin 5 derivative and its single-amino-acid variant.

**FIG 1:**
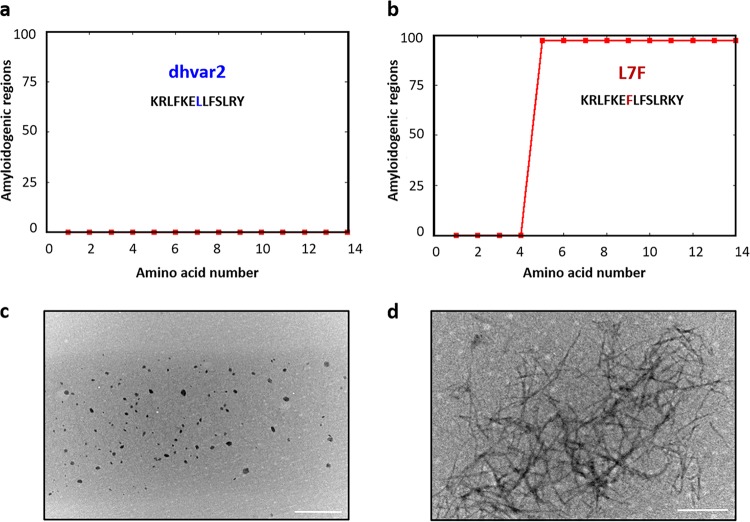
Self-assembling capabilities of an antifungal peptide and its single-amino-acid variant. (a and b) Waltz algorithim predictions of amylogenic regions in (a) dhvar2 and (b) the L7F variant. (c and d) Morphology of the assemblies (at 1 mg/ml in water) obtained via transmission electron microscopy for (c) dhvar2 and (d) L7F. Scale bar, 500 nm.

The WALTZ algorithm, which predicts amylogenic peptide regions, identified a minimal amino acid mutation within dhvar2 which was required for ordered assembly and suggested that substitution of leucine with phenylalanine at position 7 would yield a peptide with a high propensity for self-assembly (Fig. 1a and b). We tested this prediction *in vitro* via transmission electron microscopy (TEM), which demonstrated the formation of long, unbranched nanoassemblies by L7F; no ordered assemblies were observed for the unmodified dhvar2 peptide (Fig. 1c and d).

### Antifungal activity of each of the peptides.

**FIG 2:**
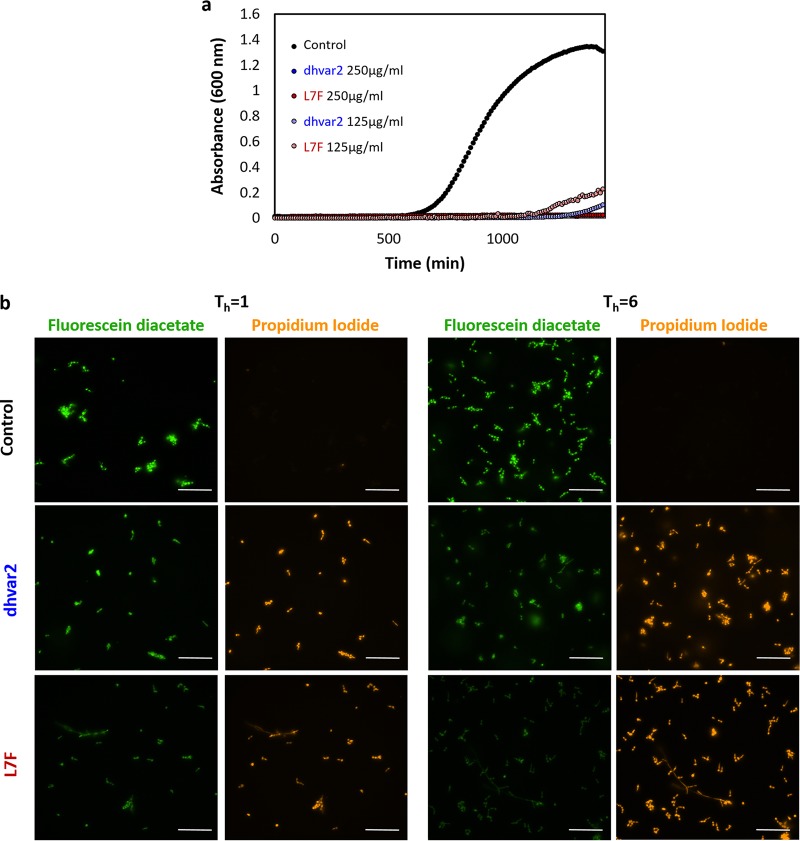
Antifungal activity and loss of cell viability due to treatment with either dhvar2 or L7F. (a) Cell growth over 24 h after addition of either peptide added at T = 0. Black, no peptide; blue, dhvar2; red, L7F, measured as OD_600_. (b) Yeast viability staining by fluorescent Live (fluorescein diacetate, green)/Dead (propidium iodide, red) reagents. Control, no peptide; dhvar2 and L7F, 250 μg/ml peptide as indicated for 1 h (left) and 6h (right). Scale bars, 50 μm.

Antifungal capabilities of these peptides were measured using standard growth curves in rich medium (yeast extract-peptone-dextrose [YPD]). Like histatin 5, both peptides inhibited fungal growth considerably at a 125 μg/ml concentration of each peptide and completely at 250 μg/ml (Fig. 2a). Fungal viability, monitored with Live/Dead staining (fluorescein diacetate and propidium iodide [PI]), revealed high levels of PI staining (a sign of cell death) as early as 1 h after addition of the peptides, with stronger PI staining of cells, including some that had undergone a yeast-to-hypha transition, at 6 h after addition of either peptide (Fig. 2b).

### Membrane interactions and their effect on fungal morphology.

**FIG 3:**
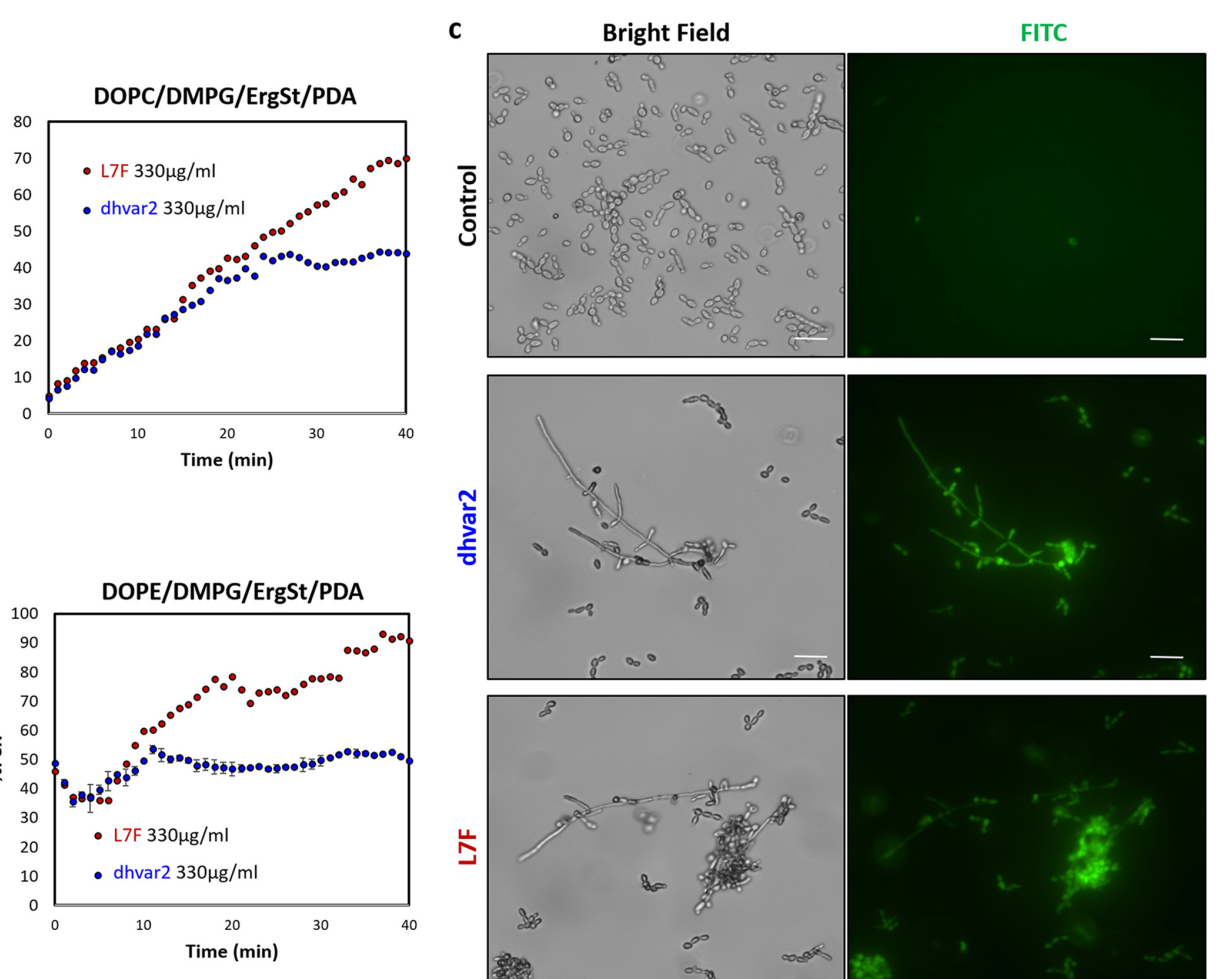
dhvar2 and L7F interact with both SUV membrane models. (a and b) Membrane interaction with the two SUV model membrane systems consisting of (a) DOPC/DMPG/ErgS/PDA and (b) DOPE/DMPG/ErgS/PDA (1:0.6:0.4:3) vesicles. PDA color transitions were induced upon peptide membrane activity. EgrSt, ergosterol. (c) Membrane permeabilization of C. albicans by AMPs. The images shown influx of FITC (53) into cells after 6 h of incubation with the indicated peptides at 250 μg/ml. FITC staining (green) indicates compromised fungal membrane integrity. Scale bar, 20 μm.

We monitored the ability of dhvar2 and LF7 to interact with model membranes by exploiting small unilamellar vesicles (SUVs) composed of phospholipids and ergosterol (ErgS). The SUVs include polydiacetylene (PDA), a colorimetric polymer that reports on the type and intensity of membrane interactions (51, 52). Two SUVs that mimic fungal membrane composition, composed of DOPC/DMPG/ErgS/PDA and DOPE/DMPG/ErgS/PDA (1:0.6:0.4:3), were utilized. Importantly, both peptides exhibited significant colorimetric responses, albeit to different extents. The response to dhvar2 was 2-fold lower than that to L7F, in both SUV types (Fig. 3a). Interestingly, the kinetic differences between the ways in which the two peptides interacted with each model membrane indicated differential modes of interaction. In the phosphatidylcholine-containing system, the signal increased gradually for both peptides, corresponding to a slow penetration into the membrane interior; dhvar2 caused less disturbance in the membrane than L7F. In the phosphatidylethanolamine (PC)-containing system, an instantaneous increase in the signal was observed, indicating that both peptides adhered to the surface immediately. A strong signal coupled with the absence of a plateau in the kinetic graphs of L7F may indicate complete destruction of the membrane.

The antimicrobial activity of most AMPs is due to membrane permeabilization following association of the peptide with the plasma membrane. To address the mechanism of dhvar2 and L7F antifungal activity in C. albicans, we measured the degree of membrane damage by the use of a fluorescein isothiocyanate (FITC)-based membrane permeation assay (53). Free FITC cannot traverse intact cell membranes and enters (and stains) cells only after their plasma membrane is damaged. Incubation with either of the peptides resulted in a marked fluorescent signal result, with 95% positive fluorescence (compared to 1% FITC fluorescence in the no-peptide control) (Fig. 3b). Thus, both peptides cause extensive membrane permeabilization.

**FIG 4:**
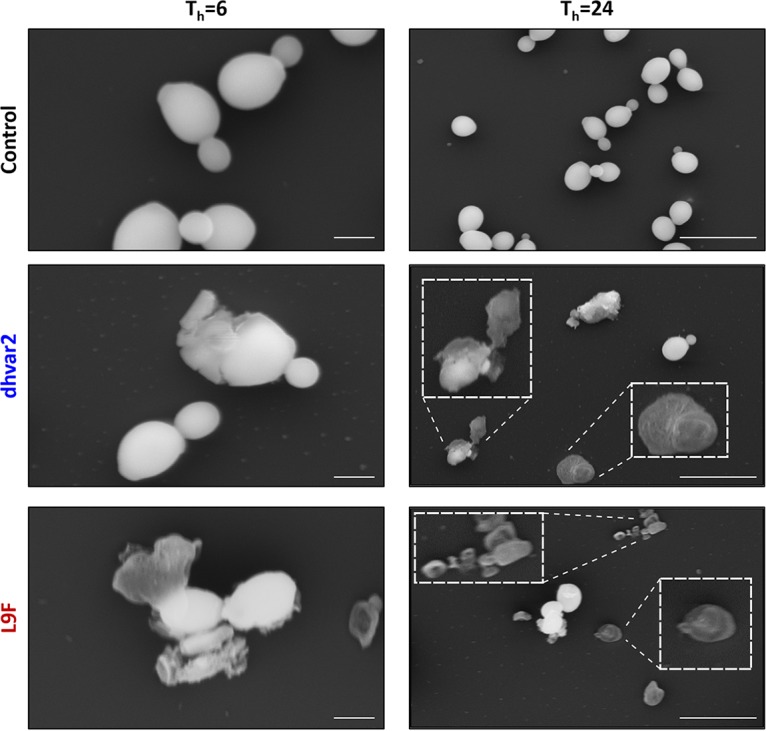
dhvar2 and L7F cause extensive cell lysis. High-resolution scanning electron microscope micrographs were collected after exposure of C. albicans to the indicated peptides (250 μg/ml) for 6 or 24 h as indicated. Scale bars, 2 μm and 10 μm (following 6 h and 24 h of treatment, respectively). Micrographs are representative of 3 independent experiments; the effect was widespread and identified in all fields evaluated (over 50 per sample).

To get a closer view of cell damage, we monitored cell integrity using scanning electron microscopy (SEM) after exposure of the cells to each of the peptides. Cell membranes had numerous nicks and tears after 6 h of exposure to either of the peptides; in many cases, the cells appeared to have lysed with contents exuded from one or more sites of cell (and likely membrane) fissure (Fig. 4). The cell damage was even more dramatic after 24 h of peptide exposure, with the few unlysed cells appearing deformed and deflated with numerous membrane perturbations (Fig. 4). Taken together, these results support the idea that membrane lysis is a major mechanism of action for both dhvar2 and L7F and that the ability to form amyloid-like assemblies (seen with L7F and not with dhvar2) is not a requirement for its membrane association, cell lysis, and antifungal activity.

### Antibacterial activity of each of the peptides.

**FIG 5:**
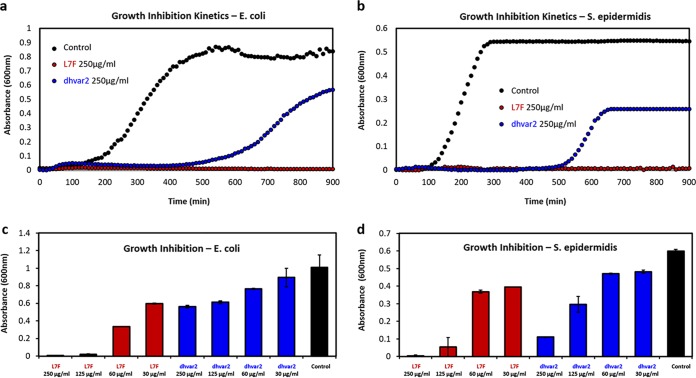
Histatin 5 derivatives exhibit antibacterial activity that is partially associated with self-assembly. Standard growth curves in rich medium over 15 h that were determined using (a) E. coli and (b) S. epidermidis illustrate the higher efficacy of L7F than on dhvar2 (both at 250 μg/ml). The peptides exhibited dose-dependent growth inhibition of (c) E. coli and (d) S. epidermidis exposed to dhvar2 (blue), L7F (red), and a no-peptide control (black).

We next asked if the correlation between antibacterial activity and self-assembly holds for these two peptides. Bacterial kinetic growth inhibition analyses were carried out for both E. coli and S. epidermidis. Interestingly, the two peptides had very different antibacterial activity levels, with L7F being 4-fold more active than dhvar2 against E. coli and 2-fold more active against S. epidermidis (Fig. 5). The growth of E. coli and S. epidermidis was completely inhibited by 125 and 250 μg/ml of L7F, respectively, and lower L7F concentrations partially inhibited growth in a dose-dependent manner (Fig. 5). While the activity of dhvar2 was lower, it also displayed dose-dependent bacterial growth inhibition (Fig. 5). These results reinforce the established connections between antibacterial activity and self-assembly.

## DISCUSSION

Therapeutic approaches that reduce the frequency of acquired resistance via new mechanisms of action have the potential to address the global problem of antimicrobial drug resistance. An emerging approach is the use of antimicrobial peptides, some of which undergo a self-assembly process that is correlated with antibacterial activities. Here, we found that a histatin 5 derivative designed to enable efficient self-assembly (L7F) had higher antibacterial activity than the related, unmodified peptide dhvar2. However, the correlation between self-assembly and antifungal activity, at least for C. albicans, did not hold. The two peptides were able to inhibit fungal growth to similar degrees, and the mechanism of action appears to be via membrane permeabilization. While one peptide self-assembled into amyloid-like fibers and the other did not (under the conditions tested), both peptides inhibited C. albicans growth, interacted with model membranes containing ergosterol, and caused dramatic damage of cell integrity that appears to have been due to cell lysis. Of course, we cannot rule out the possibility that dhvar2 can self-assemble under conditions other than those tested here.

The substitution of phenylalanine for leucine in L7F is consistent with the literature on the significance of aromaticity to self-assembly (54). Indeed, phenylalanine alone can self-assemble into ordered nanosturctures (55), and diphenylalanine nanostructures serve as a minimal model for self-assembling antimicrobial peptides (46). Interestingly, the overall antifungal activity of L7F peptide was similar to that of dhvar2, though it showed increased interactions with model membrane systems. One explanation for this apparent paradox is that the membrane activity demonstrated by dhvar2 was sufficient to elicit the fungal cell death observed; alternatively, the histatin family of peptides may affect other targets, in addition to the cell membrane. Whether the absence of correlation between self-assembly and antifungal activity for derivatives of histatin 5 is a common theme for other antifungal peptides and for other fungi remains to be determined.

## MATERIALS AND METHODS

### Sample preparation.

Lyophilized powders of each of the peptides (Genscript Biotech, USA) were dissolved in ultrapure water (BioInd, Israel) to a concentration of 1 mg/ml and heated for 50 min at 90°C, allowing the peptides to reach the monomeric state, and were then allowed to cool gradually to 25°C overnight. This treatment facilitated the self-assembly and nanostructure formation of L7F but not dhvar2.

### Transmission electron microscopy (TEM).

TEM imaging was performed by applying 10-μl samples to 400-mesh copper grids covered by a carbon-stabilized Formvar film (SPI, West Chester, PA, USA). The samples were allowed to adsorb for 2 min before excess fluid was blotted off. Negative staining was then achieved by depositing 10 μl of 2% uranyl acetate on the grid for 2 min before blotting off excess fluid. Micrographs were obtained using a Tecnai 12 electron microscope (FEI, Tokyo, Japan) operating at 120 kV.

### Fungal kinetic growth inhibition analysis.

Candida albicans (SC5341) cells were streaked from glycerol stock onto YPD agar and grown for 24 h at 30°C. Colonies were suspended in 1 ml phosphate-buffered saline (PBS) and diluted to 10^3^ cells/ml in a 96-well plate containing a gradient of 2-fold dilutions per step of each of the peptides in YPD. Kinetic growth inhibition was determined by measurements of optical density at 600 nm (OD_600_) by the use of a Tecan plate reader (Infinite F200 Pro; Tecan, Switzerland). The kinetic analysis results presented are representative of three experiments conducted independently.

### Fungal viability analysis.

Candida albicans (SC5341) cells were suspended in 1 ml PBS and diluted to 10^3^ cells/ml in a 24-well plate containing 250 μg/ml of each of the peptides in YPD. Following 1 and 6 h of incubation, the samples were washed thrice with saline solution, incubated for 15 min in a solution containing fluorescein diacetate (6.6 μg/ml) and propidium iodide (5 μg/ml), and washed with saline solution again. Fluorescence emission was detected using an Eclipse E600 fluorescence microscope (Nikon, Japan). The results presented are representative of three independent experiments.

### Interaction with model membrane systems. (i) Vesicle preparation.

Vesicles containing the diacetylene monomer 10,12-tricosadiynoic acid (TRCDA) and the lipid and ergosterol components DOPC/DMPG/ErgS/PDA and DOPE/DMPG/ErgS/PDA (1:0.6:0.4:3) were dissolved in chloroform/ethanol (1:1) and dried together *in vacuo* to reach a constant weight, followed by addition of deionized water to reach a final concentration of 1 mM, and were subsequently subjected to probe sonication at 40 W at 70°C for 3 min. The vesicle solution was subsequently cooled at room temperature and kept at 4°C overnight. The solution was then irradiated at 254 nm for 30s, resulting in intense blue color appearance due to polymerization of the diacethylene units.

### (ii) Fluorescence spectroscopy.

Fluorescence was measured on a Fluscan Ascent microplate reader using a 96-well microplate (Grainer), excitation at 485 nm and emission at 555 nm, and long-pass (LP) filters with normal slits. The background fluorescence from the vesicles alone was negligible. Samples were prepared for fluorescence measurements by adding 30 μl of each of the peptides at 1 mg/ml to 30 μl of lipid/PDA vesicles followed by addition of 30 μl 50 mM Tris-base buffer (pH = 8.0). The samples were incubated at 27°C during the measurements. Percent fluorescent chromatic responses (%FCR) were calculated according to the following formula: %FCR= [EmI/Emred] * 100% (where EmI is the value obtained for the vesicle solution after incubation with the compounds and Emred is the maximal fluorescence value obtained for the red-phase vesicles [treated with NaOH 1 M]). Results displayed are representative of three experiments conducted independently.

### FITC uptake assay.

This assay was adapted from a previously reported method (53). Candida albicans (SC5341) cells were suspended (2 × 10^7^ cells per ml) in 10 mM sodium phosphate buffer (pH 7.4) and treated with each of the peptides for 6 h at 30°C. Samples were then incubated with 6 μg/ml FITC (the stock solution is 10 mg/ml in acetone) plus 10 mM sodium phosphate buffer at 30°C for 30 min. A 10-μl volume of each sample was plated on an individual glass slide, and the slides were then washed with 10 mM sodium phosphate buffer thrice and examined using an Eclipse E600 fluorescence microscope (Nikon, Japan). The results presented are representative of three independent experiments.

### High-resolution scanning electron microscopy.

Fungal samples treated with each of the peptides for 6 and 24 h were centrifuged at 5,000 rpm for 5 min, washed thrice in PBS, and fixed in 2.5% glutaraldehyde–PBS buffer for 1 h. Samples were then washed thrice in PBS and fixed in 1% OsO_4_–PBS buffer for 1 h, followed by a dehydration series performed with ethanol. Samples were then left in absolute ethanol for 30 min and placed onto glass coverslips, followed by critical point drying and coating with gold. Micrographs were recorded using a JSM-6700F field emission scanning electron microscope (FE-SEM) (JEOL, Tokyo, Japan) operating at 10 kV. The micrographs displayed are representative of results of three experiments conducted independently.

### Bacterial kinetic growth inhibition analysis.

Each of the peptides was diluted by serial 2-fold dilutions in M9 minimal media in Corning (3879) 96-well plates (Sigma-Aldrich, Israel). E. coli bacteria (ATCC 25922) were grown overnight in M9 minimal media and diluted 1,000-fold in M9 media and grown for 5 h at 37°C. In Corning (3596) 96-well plates, 75-μl volumes of the serial 2-fold dilutions of each test compound were added to 75 μl of growth medium containing bacteria (5 × 10̂^6^ CFU/ml). Bacteria and test compounds were incubated overnight at 37°C, and kinetic growth inhibition was determined by measurements of optical density at 600 nm using a Biotek Synergy HT microplate reader. Results displayed are representative of three experiments conducted independently.
